# 

**Published:** 1904-01-16

**Authors:** 


### Publications.

JUST ISSUED. EIGHTH EDITION. Crown 8vo. **10s. 6d.**

### PHARMACY, MATERIA MEDICA AND THERAPEUTICS

It now rewritten and printed in large type, and is made a companion to the following volume. It contains an account of the action **of every drug in the B.P.**, as well as a section upon all the newest drugs, with an Introduction to the Art of Dispensing With woodcuts, prescriptions, &c

Also FOURTH EDITION. 1055 pages, 8vo. **16s.**

### DICTIONARY OF TREATMENT.

Containing a Revised and up-to-date Article upon every Diseased Condition, whether Medical Surgical, Gynæcological, or Ophthalmological, as well as Articles upon Poisoning, Drowning, and the various Surgical Emergencies and Accidents, arranged for Rapid Reference.

By W. WHITLA, M.A., M.D., Professor of Materia Medica, Queen's College, Belfast; Senior Physician to the Royal Victoria Hospital, &c.

The Publisher will supply both volumes, carriage paid, for **21/-**

London HENRY RENSHAW, 356 Strand.

### THE HANDBOOK OF THE OPEN-AIR TREATMENT

By Dr. CHARLES REINHARDT

(Visiting Physician to Hailey Sanatorium, Wallingford, and Stourfield Park Sanatorium, Bournemouth),

Contains Views, Descriptions, and Information respecting 30 Sanatoria in Great Britain.
Price: Cloth, 2/6; Paper, 1/- (postage 4d.)

The "HEALTH RESORT" Publishing Office, 379 Strand, London, W.C.

Crown 8vo. Illustrated. Price **2s. 6d.** net.

### ANÆSTHETICS. By J. BLUMFELD,

M.D.Cantab., Senior Anæsthetist to St. George's Hospital; Anæsthetist to the Grosvenor Hospital for Women and Children, London, and to the National Dental and Metropolitan Hospitals.
*Lancet.*—"....In writing this handbook the author has been quite successful." *Australasian Medical Gazette.*—"We can strongly commend this book." *Medical Chronicle.*—"The book is one which we can heartily recommend to senior students and practitioners."
London: BAILLIÈRE, TINDALL & COX, 8 Henrietta St., Covent Garden.

Second Edition. Crown 8vo., price 3/6 net.
With original "Plea—1900—for the State Registration of Nurses."

### MOCK NURSES OF THE LATEST FASHION.

By FREDERICK J. GANT, F.R.C.S.,

Consulting Surgeon to the Royal Free Hospital.

BAILLIÈRE, TINDALL & COX,

Henrietta Street, Covent Garden, London.

NOW READY. Demy 8vo. Price **5/-** net.

### ASTHMA IN RELATION TO THE NOSE.

*WITH NOTES OF 402 CASES.*

By ALEXANDER FRANCIS, M.B., B.C.Cantab

London: ADLARD & SON, Bartholomew Close, E.C.

### CHARTS.

**PRICES (Post or Carriage Paid):—**
1,000, 25s.; 500, 13s. 6d.; 250, 7s. 6d.; 100, 3s. 6d.; 50, 2s.; 25, 1s. 3d.
SPECIMENS POST FREE.

**THE BEST AND CHEAPEST PUBLISHED.**
GOULD'S TEMPERATURE CHARTS, Morning and Evening.
GOULD'S FOUR-HOUR CHARTS, Printed in Violet for hours 2, 6, 16.
GOULD'S FOUR-HOUR CHARTS, Printed in Chocolate for hours 4, 8, 12.
GOULD'S TWO-HOUR CHARTS.
BENTON'S DIET CHARTS, each Chart lasting two days.
BOARDS to hold Charts when in use, 9d. each, 8s. 6d. per dozen.
PRIVATE NURSING CHARTS, lasting 48 hours.
CHARTS for taking the Temperature of Sick Rooms.

USED IN ALMOST ALL THE PRINCIPAL HOSPITALS AND INFIRMARIES IN THE UNITED KINGDOM.

**WODDERSPOON & CO., 5 Gate Street Lincoln's Inn Fields, W.C**

### NOTICE TO CORRESPONDENTS.

All MSS., Letters, Books for Review, and other matters intended for the Editor should be addressed The Editor, "The Hospital," The Hospital Buildings, 28 & 29 Southampton Street, Strand, W.C.

The Editor cannot undertake to return rejected MS., even when accompanied by stamped directed envelope.

**Notice to Advertisers and Subscribers.**

All Advertisements, Orders for Copies of The Hospital, and Business Communications should be addressed to **THE MANAGER (Not to the Editor),** The Hospital, 28 & 29 Southampton Street, Strand, London, W.C.

The Cost of Subscription, post free, is as follows:—Per week, 3½d.; per quarter, 3s. 9d.; per half-year, 7s. 6d.; per annum, 15s. Foreign and Colonial:—Per annum, 19s. 6d., payable, in all cases, in advance.

**NOTICE.—Vol. XXXIV. of "The Hospital" (April 1903 to September 1903), in handsome cloth binding, is now on sale at the office of this Journal, price 10s. 6d. Cases for binding the Numbers contained in this Volume 2s. Index to Vol. XXXIV., 2½d., post free.**

**The Monthly part for January is now ready. Price 1s., by post, 1s. 3d.**

### BOOKS RECEIVED.

CHATTO AND WINDUS.
"Royal Guide to London Charities." By J. Lane.

H. K. LEWIS.
"The Sterilization of Urethral Instruments." By H. T. Herring.

H. J. DRAKE.
"Rudderless Ships." By Aitam.

J. AND A. CHURCHILL.

"Practice of Gynæcology." By H. Jellett.

CASSELL AND CO., LTD.
"A Manual of Operative Surgery." Two vols. By Sir Frederick Treves, Bart.

FOOD AND COOKERY PUBLISHING AGENCY.
"Cookery for Invalids." By G. H. Senn.

BAILLIÈRE, TINDALL AND COX.
"Dictionary of Hygiene." By C. T. Kingzett and D. Homfray.

E. LLOYD, LTD.
"London Manual." By R. Donald.
"Cancer: Nature's own and only Remedy." By Carlo Carillo.

HENRY FROUDE.
"Poems." By J. Keats (with portrait).

JOHN DICK.
Dick's Penny Stories (6).—(1) Riddles; (2) Toasts and Speeches; (3) Indoor Games; (4) Puzzles; (5) Charades for Amateurs; (6) American Humour.

HIS MAJESTY'S STATIONERY OFFICE.
"Arsenical Poisoning." Final report.
"Census of England and Wales." (Index.)

CLAYE, BROWN AND CLAYE.
"A Few Facts and Figures concerning Vaccination." By J. H. Marsh.

J. WILLING, JUN., LIMITED.
"Willing's Press Guide, 1904."

### THE STUDENT OF MEDICINE.

### APPOINTMENTS.

**D. J. M. Bone**, M.D.Edin., District Medical Officer of the Ulverstone Union. **W. Victor Bonney**, M.S., M.D.Lond., F.R.C.S., M.R.C.P., Tutor in Practical Midwifery in the Middlesex Hospital Medical School. **Thos. Brown**, M.B., Ch.B.Vict., Resident Medical Officer to the Leeds Public Dispensary. **J. Paul Bush**, C.M.G., M.R.C.S.Eng., Medical Officer to the Bristol Post Office. **John Maclean Carvell**, M.R.C.S.Eng., L.S.A.Lond., Registrar to Out-patients, Central London Throat and Ear Hospital. **George Perry Duprey**, M.R.C.S., L.R.C.P., L.D.S., Dental Superintendent to the Royal Dental Hospital of London, Leicester Square. **G. B. Howe**, M.R.C.S., L.S.A., J.P., Medical Officer No. 5 District, and Public Vaccinator Nos. 3 and 5 Districts of the Ashton-under-Lyne Union. **S. T. Irwin**, M.B., Ch.B., Junior House Surgeon, St. Peter's Hospital for Stone, Henrietta Street, W.C. **L. A. W. Johnston**, M.R.C.R., L.R.C.P., District Medical Officer of the Birmingham Union. **Hugh McManus**, M.B., Second Resident Medical Officer of the Nottingham Union Workhouse. **D. W. Main**, M.B., Ch.B.Vict., Medical Officer of Health for the Bollington Urban District, and District Medical Officer of the Macclesfield Union. **F. H. Moore**, L.R.C.P.&S.Irel., District Medical Officer of the Dunmow Union. **J. E. Passmore**, M.R.C.S., L.R.C.P.Lond., District Medical Officer of the Basingstoke Union. **W. R. Penny**, M.R.C.S., L.R.C.P.Lond., District Medical Officer of the Ulverstone Union. **K. O. Vaughan**, M.B.Lond., Superintendent, Victoria Dufferin Hospital, Calcutta. **C. W. Von Bergen**, M.B., B.S.Durh., Medical Officer for the Ashstead District of the Epsom Union.

### "The Hospital" Institutional Library.

"Hospitals and Asylums of the World." 4 Vols., with a Portfolio of Plans, complete £12 12s.

"Burdett's Hospitals and Charities, 1903." 5s. net.

"Burdett's Official Nursing Directory, 1903." 3s. net.

"Cottage Hospitals, General, Fever, and Convalescent." 10s. 6d.

"Uniform System of Accounts for Hospitals and Public Institutions." New Edition. 4s. net.

"Hospital Expenditure: The Commissariat." 2s. 6d.

"A Legal Handbook for the Use of Hospital Authorities." 2s. 6d.

"The Cottage Hospital Case Book or Register of Patients." 10s. and 12s. 6d.

All these are published by the SCIENTIFIC PRESS, Ltd., and may be obtained through any bookseller, or direct from the publishers, 28 and 29 Southampton Street, Strand, London, W.C.

### Books on the Care of the Young.

### The Mother's Help and Guide to the Domestic Management of her Children.

By Dr. MURRAY BRAIDWOOD.

Price 2/- post free.

Useful in health and illness.

### Cleanliness in Children.

By ROSE PETTY.

Price 2d. post free.

Will aid the School Nurse in her mission.

### Sight and Hearing in Childhood.

By R. BRUDENELL CARTER, F.R.C.S., and ARTHUR CHEATLE, F.R.C.S.

Price 2/- net, post free 2/3.

This is a most useful and important work by two eminent authorities.

### Spinal Curvature and Awkward Deportment.

By Dr. GEORGE MÜLLER.

Price 2/6 post free.

This is a book which should be in the hands of all who have the care of children, as it points out both prevention and cure in simple language.

### Some Health Aspects of Education.

By Dr. PERCY LEWIS.

Price 1/- post free.

### Home Nursing of Sick Children.

By J. D. E. MORTIMER, F.R.C.S.

Price 1/- post free.

A most useful little book, full of practical hints on a difficult subject.

### The Physiological Feeding of Infants.

By Dr. ERIC PRITCHARD.

Price 1/- post free.

A most useful guide to artificial feeding.

*These books are obtainable of all Booksellers, and are published by* THE SCIENTIFIC PRESS, LIMITED, 28 & 29 Southampton Street, Strand, London, *who will send their latest Catalogue of Nursing Text-books post free to any Nurse on application.*

### Two Important Text-Books.

### ELEMENTARY ANATOMY AND SURGERY FOR NURSES.

By WILLIAM McADAM ECCLES, M.S.Lond., M.B., F.R.C.S., L.R.C.P.

Crown 8vo., profusely illustrated, cloth, **2/6** post free.

"The book will be valuable to nurses who are beginning the study of anatomy. The instruction is given very clearly and concisely, and the read r is able to glean much information in a very condensed form."—*Nursing Notes.*

### ELEMENTARY PHYSIOLOGY FOR NURSES.

By C. F. MARSHALL, M.D., B.Sc., F.R.C.S.

Crown 8vo., illustrated, cloth, **2/-** post free.

"Nurses will find in it all that they require to know of the subjects treated."—*Daily Chronicle.*

*To be obtained of all Booksellers, or of*
THE SCIENTIFIC PRESS, LIMITED,
28 & 29 Southampton Street, Strand, London, W.C.

